# Efficacy of urapidil for the treatment of patients with senile hypertension and acute heart failure

**DOI:** 10.1097/MD.0000000000017352

**Published:** 2019-10-11

**Authors:** Yan-Zhong Xie, Jian-Ming Ni, Shan-Jing Zhang, Guo-Rong Ding, Jun-Fei Feng

**Affiliations:** a Department of Emergency,; b Department of Respiratory Medicine, Hangzhou Fuyang Hospital of Traditional Chinese Medicine, Hangzhou, China.

**Keywords:** acute heart failure, efficacy, safety, senile hypertension, urapidil

## Abstract

**Background:**

Previous clinical studies have reported that urapidil can effectively treat patients with senile hypertension (SH) and acute heart failure (AHF). However, no studies have systematically assessed the efficacy and safety of urapidil for patients with SH and AHF. Thus, this study will investigate the efficacy and safety of urapidil for SH and AHF.

**Methods:**

In this study, we will search the following electronic databases from inception to the June 30, 2019: MEDLINE, EMBASE, Cochrane Library, Google scholar, Springer, WANGFANG, and China Knowledge Resource Integrated Database. We will search all these electronic databases without language limitations. We will also search grey records to avoid missing potential literature. In this study, only randomized controlled trials on assessing efficacy and safety of urapidil for SH and AHF will be considered. We will use RevMan 5.3 software and STATA 15.0 software to carry out statistical analysis.

**Results:**

This study will evaluate the efficacy and safety of urapidil for SH and AHF by assessing all-cause mortality, change in body weight, urine output, change in serum sodium; and incidence of all adverse events.

**Conclusion:**

This study will provide latest evidence of the efficacy and safety of urapidil for patients with SH and AHF.

**Dissemination and ethics:**

This study will only analyze published data; therefore, no ethical approval is needed. The findings of this study will be published at peer-reviewed journals.

**Systematic review registration:**

PROSPERO CRD42019139344.

## 1 Introduction

Acute heart failure (AHF) is a very common disorder in the clinic practice of cardiovascular department.^[1–2]^ It is characterized by an acute deterioration in cardiac function.^[3–6]^ In addition, such severe disorder has been a major public health problem, and is also associated with high intervention and health care costs, as well as high risk of mortality and morbidity.^[7–10]^ It has been estimated that its prevalence is about 4.2 million in China and 23 million globally.^[11–13]^ Previous studies have reported that patients with senile hypertension (SH) are more likely to suffer from AHF among elderly population.^[14–19]^ Therefore, it is very important to manage patients with SH and AHF. Fortunately, urapidil has reported to treat patients with SH and AHF very well.^[20–23]^ However, there is still no systematic study to assess the efficacy and safety in patients with SH and AHF. Therefore, this study will firstly explore the efficacy and safety of urapidil for the management of SH and AHF.

## 2 Methods

### 2.1 Objective

This study will systematically assess the efficacy and safety of urapidil for patients with SH and AHF.

### 2.2 Study registration

We have registered this study on PROSPERO (CRD42019139344). We have reported this study according to the statement of Preferred Reporting Items for Systematic Reviews and Meta-Analysis Protocol.^[24]^

### 2.3 Criteria for considering studies for this study

#### 2.3.1 Types of studies

We will include randomized controlled trials (RCTs) which have explored the efficacy and safety of urapidil for patients with SH and AHF.

#### 2.3.2 Types of participants

All patients with SH and AHF will be included regardless the race, sex, and age.

#### 2.3.3 Types of interventions

Patients in the experimental group can receive any forms of urapidil.

Patient in the control group can receive any interventions, except urapidil.

#### 2.3.4 Type of outcome measurements

The primary outcome consists of all-cause mortality. The secondary outcomes comprise of change in body weight, urine output, change in serum sodium; and incidence of all adverse events.

### 2.4 Search methods for identification of studies

#### 2.4.1 Electronic searches

For this study, we will search the following electronic databases from inception to the June 30, 2019: Cochrane Library, MEDLINE, EMBASE, Google scholar, Springer, WANGFANG, and China Knowledge Resource Integrated Database without language restrictions. Details of the search strategy for Cochrane Library are showed in the Table 1. We will also use identical search strategies to other electronic databases.

**Table 1:**
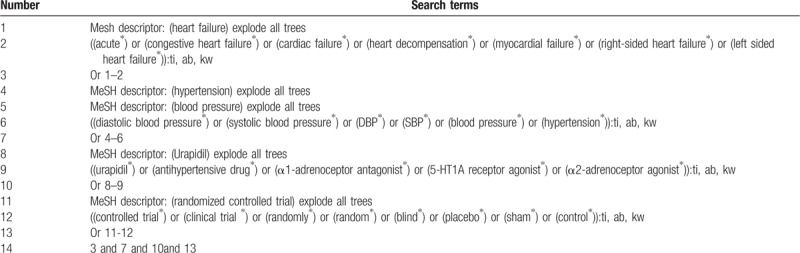
Search strategy for Cochrane Library database.

#### 2.4.2 Search for other resources

We will also check unpublished studies, dissertations, and reference lists of obtained reviews in this study.

### 2.5 Data collection and analysis

#### 2.5.1 Selection of studies

**Figure 1:**
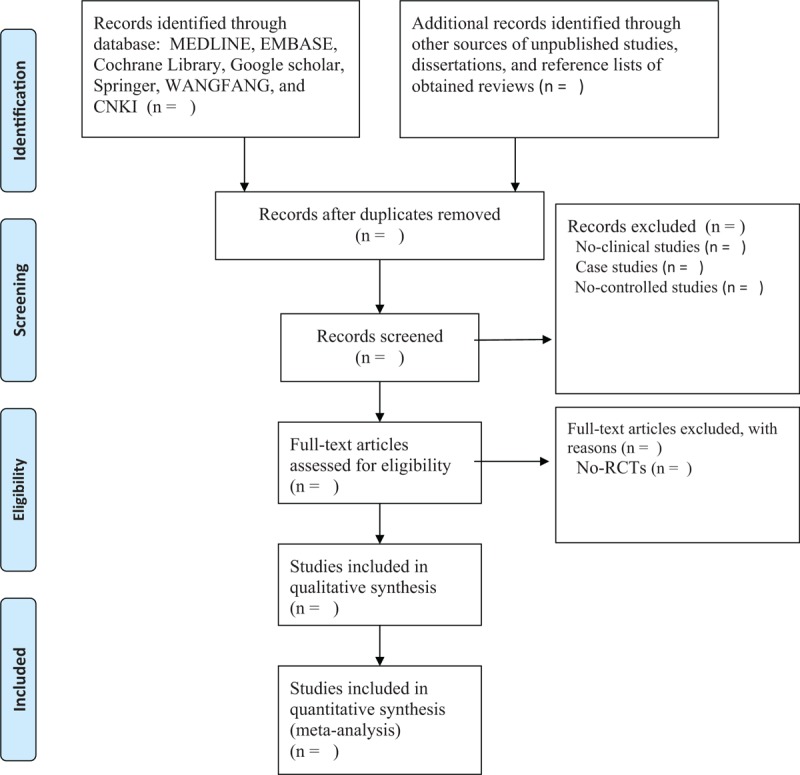
Process of study selection.

Two independent authors will check titles and abstracts of all records according to the pre-designed eligibility. A third experienced author will be invited to solve disagreements between 2 authors by discussion. Initially, all searched studies will be checked if they meet the primary inclusion criteria. Then, all irrelevant studies will be excluded, and remaining studies will be read by full-texts. The process of study selection will be reported in the diagram chart in Figure 1.

#### 2.5.2 Data extraction and management

Two authors will independently extract and summarize details of eligible studies by using previous designed data extraction form. A third author will help to solve any disagreements between 2 authors. The following information will be extracted.

(1) Study characteristics, such as title, author, published year, etc;
(2) Patient characteristics, such as race, age, gender, etc;
(3) Study design and methods, such as sample size, randomization, blinding, etc;
(4) Intervention details, such as treatments, comparators, duration, etc;
(5) Outcome measurements, such as primary, secondary outcomes, safety, etc.

#### 2.5.3 Dealing with missing data

We will attempt to obtain any missing or insufficient data by contacting primary authors. If we cannot require those additional data, only available data will be analyzed in this study.

#### 2.5.4 Assessment of risk of bias

Two independent authors will evaluate the risk of bias for the included studies. We will use Cochrane risk of bias tool. Any divisions between 2 authors will be solved by a third author through discussion. It has seven criteria, each 1 is further judged with low risk of bias, unclear risk of bias and high risk of bias.

### 2.6 Statistical analysis

#### 2.6.1 Measurement of treatment effect

For each trial with dichotomous outcomes, we will calculate as odd ratio or risk ratio and 95% confidence intervals. Where outcomes for continuous outcomes, we will calculate as mean difference or standardized mean difference and 95% confidence intervals.

#### 2.6.2 Assessment of heterogeneity

We will apply *I*^2^ statistic to check heterogeneity among trials. *I*^2^ ≤ 50% indicates reasonable heterogeneity, and a fixed-effect model will be applied. *I*^2^ > 50% indicates substantial heterogeneity, and a random-effect model will be used.

#### 2.6.3 Data synthesis

When the reasonable heterogeneity is obtained, meta-analysis will be carried out. When the substantial heterogeneity is identified, subgroup analysis will be performed to check any reasons that may lead to such high heterogeneity. If significant heterogeneity is still obtained after subgroup analysis, we will not pool the data and will only report narrative summary.

#### 2.6.4 Subgroup analysis

In this study, subgroup analysis will be performed in accordance with the different treatments, controls, and outcomes.

#### 2.6.5 Sensitivity analysis

We will carry out sensitivity analysis to check the robustness and stability of outcome results by removing low methodological quality studies.

#### 2.6.6 Reporting bias

We will check reporting bias using funnel plot and Egger test^[25]^ when more than 10 trials are entered in this study.

## 3 Discussion

Previous studies have reported that urapidil is used for the treatment of patients with SH and AHF.^[20–23]^ However, no systematic review has explored the efficacy and safety of urapidil for treating SH and AHF. Thus, this study will demonstrate that urapidil administration is as effective in patients with SH and AHF. In this study, we will search all relevant studies without language limitations, and all potentials studies related to the urapidil for treating SH and AHF will be considered. The results of this study will summarize update evidence on the efficacy and safety of urapidil for treating SH and AHF. It may also provide helpful evidence for patients, clinician, and health policy-makers.

## Author contributions

**Conceptualization:** Yan-Zhong Xie, Jian-Ming Ni, Shan-Jing Zhang, Guo-Rong Ding.

**Data curation:** Yan-Zhong Xie, Jian-Ming Ni, Guo-Rong Ding.

**Formal analysis:** Yan-Zhong Xie, Jian-Ming Ni, Shan-Jing Zhang.

**Investigation:** Guo-Rong Ding.

**Methodology:** Yan-Zhong Xie, Jian-Ming Ni, Shan-Jing Zhang.

**Project administration:** Guo-Rong Ding.

**Resources:** Yan-Zhong Xie, Jian-Ming Ni, Shan-Jing Zhang.

**Software:** Yan-Zhong Xie, Jian-Ming Ni, Shan-Jing Zhang.

**Supervision:** Guo-Rong Ding.

**Validation:** Yan-Zhong Xie, Jian-Ming Ni, Shan-Jing Zhang, Guo-Rong Ding.

**Visualization:** Yan-Zhong Xie, Jian-Ming Ni, Guo-Rong Ding.

**Writing – original draft:** Yan-Zhong Xie, Jian-Ming Ni, Shan-Jing Zhang, Guo-Rong Ding.

**Writing – review & editing:** Yan-Zhong Xie, Jian-Ming Ni, Shan-Jing Zhang, Guo-Rong Ding.
